# Efficacy of red yeast rice extract on myocardial infarction patients with borderline hypercholesterolemia: A meta-analysis of randomized controlled trials

**DOI:** 10.1038/s41598-020-59796-5

**Published:** 2020-02-17

**Authors:** Bunleu Sungthong, Chenchira Yoothaekool, Sornsalak Promphamorn, Wiraphol Phimarn

**Affiliations:** 1 Pharmaceutical Chemistry and Natural Products Research Unit, Faculty of Pharmacy, Mahasarakham University, Kantharawichai, Maha Sarakham 44150 Thailand; 2 Social Pharmacy Research Unit, Faculty of Pharmacy, Mahasarakham University, Kantharawichai, Maha Sarakham 44150 Thailand

**Keywords:** Lipoproteins, Interventional cardiology

## Abstract

Red yeast rice (RYR) extract is widely used for improving cardiovascular outcomes and lipid profiles. However, RYR efficacy on cardiovascular outcomes in myocardial infarction (MI) patients remains unclear. This meta-analysis assessed efficacy of RYR extract in MI patients with borderline hypercholesterolemia. PubMed, CENTRAL, CINAHL, Scopus, Web of Science, and Clinicaltrials.gov were systematically searched from inception through May 2019 for relevant publications. Seven studies with 10,699 MI patients diagnosed with borderline hypercholesterolemia were included. Follow-up periods ranged from 4 weeks – 4.5 years and the studies were overall of high quality with low risk of bias. RYR extract (1,200 mg/day) reduced nonfatal MI (risk ratio (RR) = 0.42, 95% CI 0.34 to 0.52), revascularization (RR = 0.58, 95% CI 0.48 to 0.71), and sudden death (RR = 0.71, 95% CI 0.53 to 0.94). RYR extract also lowered LDL (weighted mean difference (WMD) = −20.70 mg/dL, 95% CI −24.51 to −16.90), TC (WMD = −26.61 mg/dL, 95% CI −31.65 to −21.58), TG (WMD = − 24.69 mg/dL, 95% CI −34.36 to −15.03), and increased HDL levels (WMD = 2.71 mg/dL, 95% CI 1.24 to 4.17). This meta-analysis indicated that RYR extract in MI patients with borderline hypercholesterolemia is associated with improved cardiovascular outcomes and lipid profiles.

## Introduction

Cardiovascular diseases (CVD), including cerebrovascular disease, coronary heart disease, and peripheral arterial diseases, are the main burden disease in the world^1^. Previous studies has indicated that several dietary factors such as high sodium consumption and high fat diet have been associated with a higher risk of CVD^2^. Data from a recent study suggested that lower levels of blood cholesterol reduced the risk of major vascular events, and lower low density lipoprotein cholesterol (LDL-C) levels were associated with reduced rates of major coronary events^3^. Patients with borderline hypercholesterolemia, a healthy lifestyle modification, healthy diet, physical activity and weight control, is recommended to reduce the risk of artherosclerotic cardiovascular disease (ASCVD). Patients with clinical ASCVD, statins are the first line therapy to reduce LDL-C. In patients with high risk of ASCVD or severe primary hypercholesterolemia, statin combination with ezetimibe and/or PCSK9 inhibitor may be considered^4^.

Red yeast rice (RYR) is processed by fermenting white rice with the yeast *Monascus purpureus*, producing rice that is red in color^5^. RYR contains monacolin K, a fungal secondary metabolite that is structurally similar to natural statins. The primary mechanism of action of monacolin K is inhibition of the key enzyme, 3-hydroxy-3-methylglutaryl coenzyme A (HMG-CoA) reductase, involved in cholesterol synthesis^6^. In many countries, various RYR products are available on the market as a food supplement. Several clinical trials have evaluated the association between RYR consumption and dyslipidemia, ischemic heart disease, and cardiovascular disease^7–10^. While a previous meta-analysis showed that RYR extract had beneficial effects for hyperlipidemia patients, quantitative analyses on the effect of RYR extract on cardiovascular outcomes is still limited. One systematic review of 22 trials took a more comprehensive search strategy, utilizing the international Data Base (IDB)^11^. In that review, the authors included studies that examined RYR in coronary heart disease that was complicated by dyslipidemia. The results indicated that RYR extract exhibited a positive effect on lipid profiles. However, recommendations resulting from previous studies were inconclusive. Nevertheless, there were several important limitations to this systematic review. The main limitations were the small sample sizes and the small number of included trials that assessed cardioprotective effects. Moreover, there was no systematic review and meta-analysis in MI patients with borderline hypercholesterolemia. For this reason, we conducted a systematic review and meta-analysis of relevant Randomized Control Clinical Trials (RCT) that aimed to evaluate the efficacy and safety of RYR on MI patients with borderline hypercholesterolemia.

## Methods

The Cochrane Collaboration framework guidelines was used to conducted this systematic review^12^. The report follows the Preferred Reporting Items for Systematic Reviews and Meta-Analyses (PRISMA) statement^13^.

### Search strategies and study selection

The original articles were searched by comprehensive electronic database: PubMed, Cochrane Central Register of Clinical Trial (CENTRAL), CINAHL, Scopus, Web of Science, and Clinicaltrials.gov. The search did not impose any date or language restrictions; databases were searched from their inception through May 2019. Strategic search terms included (“Red Yeast Rice” OR “RYR”) AND “Myocardial Infarction (MeSH)” OR (“lipid profile” OR “lipid lowering”) OR “non-fatal” OR “fatal” AND “randomized controlled trial”. References included in the papers selected for full text review were also scanned to identify potential studies that were not indexed in the databases listed above.

Research articles were included if they were RCTs investigating the clinical effects of RYR extract formulations on participants with MI and borderline lipid profile levels. We excluded studies performed with RYR mixed with another drug or medicinal plant.

All titles and abstracts were screened for inclusion-exclusion criteria. Two researchers (SP, CY) then independently assessed the full-text articles that potentially qualified for inclusion. Disagreements between the reviewers were resolved by discussions with WP.

### Data extraction and quality assessment

All data were independently extracted by SP and CY using a standardized extraction form. The following information was sought from each article: author, year of publication, type of study design, patient and intervention characteristics, sample size, duration of therapy, and outcome measurements.

Studies included in this review were assessed for methodological quality by SP and CY using a Jadad’s scale and the Cochrane Risk of Bias tool. The Jadad scoring system provided guidelines for preliminarily evaluation of the methodological approach of the RCT. Five items of a RCT were taken into account: (1) statement of randomization, (2) appropriateness of generating a randomized sequence, (3) use of double-blinding, (4) description of double-blinding method, and (5) details of withdrawals and dropouts. Studies that met at least three out of the five criteria were classified as high quality. The Cochrane Risk of Bias tool^14^ which contained 5 domains: bias arising from the randomization process, bias due to deviations from intended interventions, bias due to missing outcome data, bias in measurement of the outcome, and bias in selection of the reported result. The overall risk of bias for each study was classified as “low risk of bias” (low risk of bias for all domains), “some concern” (some concerns in at least one domain and no high risk of bias in any domain), or “high risk of bias” (high risk of bias in at least one domain or some concerns for multiple domains in a way that substantially lowers confidence in the results).

Disagreements between the reviewers were settled through discussion and consensus after consulting a third party (WP). Where data were missing, reviewers attempted to contact the authors to obtain the desired information.

### Outcome measures and statistical analyses

The primary outcomes were cardiovascular outcomes, defined as nonfatal MI, fatal MI, revascularization, and sudden death. The secondary outcomes included lipid lowering effects and adverse events (AE). Pooled effects were calculated and stratified according to outcomes data. Summary statistics of dichotomous outcomes were expressed as a risk ratio (RR) with 95% confidence (CI), whereas summary statistics of continuous outcomes were expressed as weighted mean difference (WMD). Statistical heterogeneity between studies was assessed using the chi-squared test and *I*^2^. A significant difference for the heterogeneity test was considered when P < 0.05, and substantial heterogeneity was reported when *I*^2^ was 50% or greater^15^. If evidence of high heterogeneity was present, we attempted to explore the underlying cause for it; subgroup analyses were performed when possible. The random effects model was used if the included studies were heterogeneous; alternatively, the fixed effects model was used if homogeneity was found. Publication bias was evaluated using a funnel plot for a particular outcome^16^. Publication bias was assessed using Egger weighted regression statistics and a visual inspection of funnel plots^17^. The statistical analysis was undertaken with Review Manager (Revman^®^) version 5.3 (Cochrane Collaboration) and STATA software version 14.

### Sensitivity and subgroup analyses

To ensure robustness of results, sensitivity analysis was performed using the one-study removal (leave-one-out) approach^18^. In addition, we performed subgroup analyses based on duration of treatments.

## Results

### Study selection

**Figure 1:**
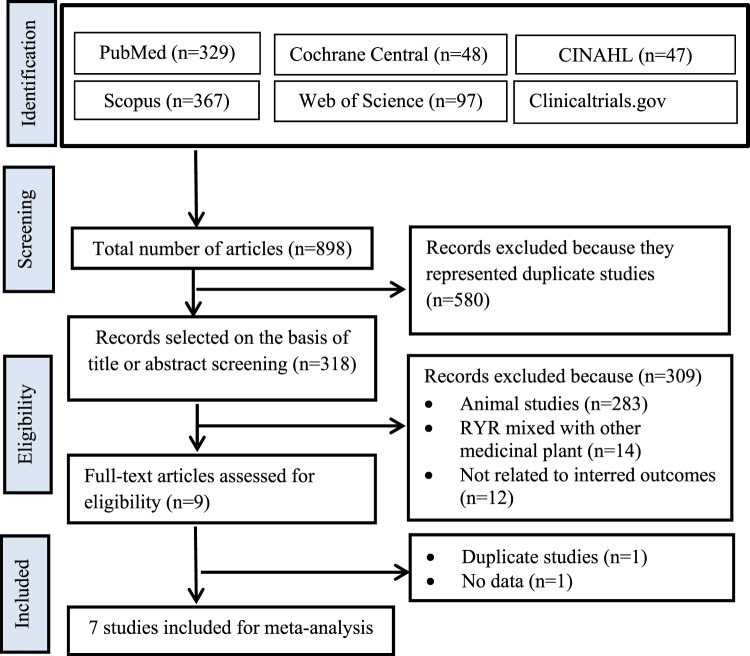
A PRISMA flow diagram describing the selection process for identifying included studies.

The PRISMA flow diagram of studies is shown in Fig. 1. The 898 related articles were identified through database searching. Upon removal of duplicate articles, 318 articles were eligible for screening. Following a rigorous screening of titles and abstracts, nine articles were selected for full text review. A total of two articles were excluded after full text review; one article represented a duplicate and the second article was lacking data. Therefore, seven articles^8,19–24^ were included in our study.

### Characteristics and methodological quality of included studies

The characteristics and methodological quality of included studies are summarized in Table 1. All of the seven selected studies were conducted in China between 2004 and 2010. Most of the included studies (6/7) were double blind randomized controlled trials; four of them had an enrolled sample size of more than 1,000 patients. The total number of participants was 10,699. The included patients ranged from 50 to 70 years old. In all studies, RYR extract was administered orally at 1,200 mg/day as an adjunctive treatment. All of the MI patients were treated by physicians who followed clinical practice guidelines that indicated prescription of pharmacological agents, including angiotensin-converting-enzyme inhibitors, calcium channel blockers, beta blockers, nitrate, aspirin, and diuretic drugs. The treatment courses ranged from 4 weeks to 4.5 years. With regard to the methodological quality of the included studies, most of them (6/7; 85.71%) were rated as high quality with a low risk of bias. Three studies did not report information concerning investigator blinding (Table 2).

**Table 1:** Characteristics of the included studies.

Authors, Year	Design	Sample size: Intervention / control	Age range (years): Intervention / control	AEs Report	Treatment duration	Intervention		Outcome measure	Jadad Score
						Treatment group	Control group		
Zhao, 2004^24^	DRCT	25/25	59.1 ± 6.3/58.2 ± 4.2	No	6 weeks	RYR 600 mg BID	Placebo capsules BID	Lipid profile	4
Hu, 2006^19^	DRCT	25/25	55.3 ± 3.4/54.1 ± 3.6	No	6 weeks	RYR 600 mg BID	Placebo capsules BID	Lipid profile	4
Li, 2009^20^	DRCT	772/758	66 ± 4.0/66 ± 4.0	Yes	4.5 years	RYR 600 mg BID	Placebo capsules BID	Lipid profile, CHD event	5
Lu, 2008^8^	DRCT	2,429/2,441	62.6 ± 7.4/58.0 ± 9.7	Yes	4.5 years	RYR 600 mg BID	Placebo capsules BID	Lipid profile, CHD event	5
Zhao, 2003^23^	RCT	25/25	58.6 ± 5.7/57.9 ± 5.7	No	6 weeks	RYR 600 mg BID	Placebo capsules BID	Lipid profile	3
Ye, 2007^22^	DRCT	735/710	69.2 ± 2.9/69.1 ± 3.0	Yes	4 years	RYR 600 mg BID	Placebo capsules BID	CHD event	5
Li, 2010^21^	DRCT	1363/1341	63.0 ± 7.1/59.2 ± 9.5	Yes	4.5 years	RYR 600 mg BID	Placebo capsules BID	Lipid profile, CHD event	5

Remark: DRCT, double blind randomized controlled trial; RCT, randomized controlled trial; AE, adverse events; CHD, coronary heart disease.

**Table 2:** Risk of bias.

Studies	Sequence generation	Allocation concealment	Investigator blinding	Patients blinding	Incomplete outcomes data	Selective outcome reporting	Other source of bias	Overall risk of bias
Zhao, 2004^24^	Low	Low	Unclear	Low	Low	Low	Low	Low
Hu, 2006^19^	Low	Low	Unclear	Low	Low	Low	Low	Low
Li, 2009^20^	Low	Low	Low	Low	Low	Low	Low	Low
Lu, 2008^8^	Low	Low	Low	Low	Low	Low	Low	Low
Zhao, 2003^23^	Low	Low	Unclear	Unclear	Low	Low	Low	Low
Ye, 2007^22^	Low	Low	Low	Low	Low	Low	Low	Low
Li, 2010^21^	Low	Low	Low	Low	Low	Low	Low	Low

### Clinical therapeutic efficacy

#### Primary outcomes

**Figure 2:**
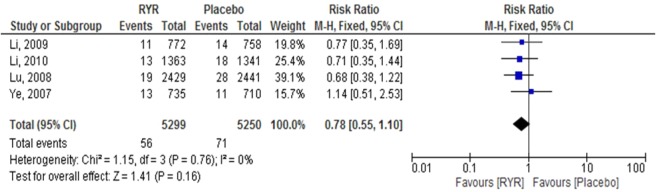
The effect of RYR extract on fatal MI.

Fatal MI. Four trials involving a total of 10,549 patients reported clinical therapeutic efficacy of RYR extract on fatal MI outcomes^8,20–22^. The pooled effect from meta-analysis demonstrated that RYR decreased the incidence of fatal MI, but there was no significant difference between RYR and placebo (RR = 0.78, 95% CI 0.55 to 1.10, P = 0.16). There was no evidence of heterogeneity among studies (I^2^ = 0.0%, P = 0.76) (Fig. 2).

**Figure 3:**
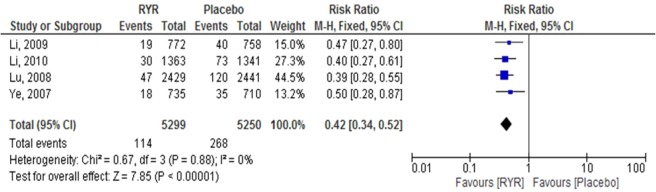
The effect of RYR extract on nonfatal MI.

Nonfatal MI. Four trials involving a total of 10,549 patients investigated the effect of RYR extract on the reduction of nonfatal MI events, compared with placebo^8,20–22^. These studies were included in our meta-analysis. The incidence of nonfatal MI was significantly reduced in the RYR-treated groups, compared with the placebo group (RR = 0.42, 95% CI 0.34 to 0.52, P < 0.00001). Heterogeneity was not observed in this outcome (I^2^ = 0.0%, P = 0.88) (Fig. 3).

**Figure 4:**
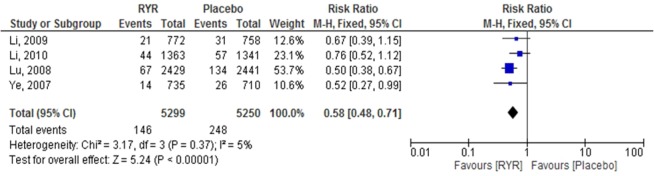
The effect of RYR extract on revascularization.

Revascularization. Revascularization included percutaneous coronary intervention (PCI) and coronary artery bypass graft (CABG). Four studies^8,20–22^ with a total of 10,549 patients reported on revascularization. The pooled results indicated that the effect of RYR extract on revascularization was significantly different from the control group (RR = 0.58, 95% CI 0.48 to 0.71, P < 0.00001). There was no evidence of heterogeneity (I^2^ = 5.00%, p = 0.37) (Fig. 4).

**Figure 5:**
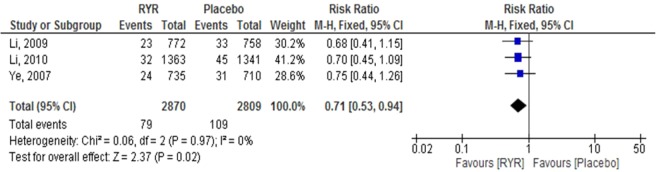
The effect of RYR extract on sudden death.

Sudden death. Three studies^20–22^.involving a total of 5,679 patients reported the incidence of sudden death. The pooled RR showed that RYR extract significantly decreased the incidence of sudden death, compared to the control group (RR = 0.71, 95% CI 0.53 to 0.94, P = 0.02). Heterogeneity was not observed between studies (I^2^ = 0.0%, P = 0.97) (Fig. 5).

### Secondary outcomes

Data from six trials indicated that the effect of RYR extract on lipid profile was superior to placebo with regard to levels of LDL (WMD, −20.70 mg/dL; 95% CI −24.51 to −16.90), Total cholesterol (TC) (WMD, −26.61 mg/dL; 95%CI −31.65 to −21.58), and Triglyceride (TG) (WMD, −24.69; 95% CI −34.36 to −15.03). Moreover, the RYR extract increased high density lipoprotein (HDL) significantly (WMD, 2.71; 95% CI 1.24 to 4.17). A statistically significant heterogeneity was detected in these four outcomes (Table 3).

**Table 3:** Meta-analysis of effects of RYR extract on all outcomes.

Outcomes (References)	No. of studies	Outcome difference		Heterogeneity	
		Mean (95% CI)	P value	I^2^	P value
LDL^8,19–21,23,24^	6	−20.70 mg/dL (−24.51 to −16.90)	<0.00001	80%	<0.00001
HDL^8,19–21,23,24^	6	2.71 mg/dL (1.24 to 4.17)	<0.00001	72%	0.0003
TC^8,19–21,23,24^	6	−26.61 mg/dL (−31.65 to −21.58)	<0.00001	90%	<0.00001
TG^8,19–21,23,24^	6	−24.69 mg/dL (−34.36 to −15.03)	<0.00001	85%	<0.00001

### Adverse events

Safety outcomes were reported in 4/7 studies, involving a total of 10,549 patients^8,20–22^. The number of AE was comparable for the RYR extract-treated and control groups. There were no reports of serious AE from RYR products following oral administration at a dose of 1,200 mg/day for 4 weeks − 4.5 years. Three studies^20–22^ reported allergic reaction, gastrointestinal discomfort, and myalgia in the RYR treatment group. In addition, two studies^21,22^ reported erectile dysfunction. No deaths were reported in any of the studies during the study periods. Only one study^8^ noted an increase in liver and renal function indicators following administration of RYR products, but these increases were not statistically significant.

### Publication bias

**Figure 6:**
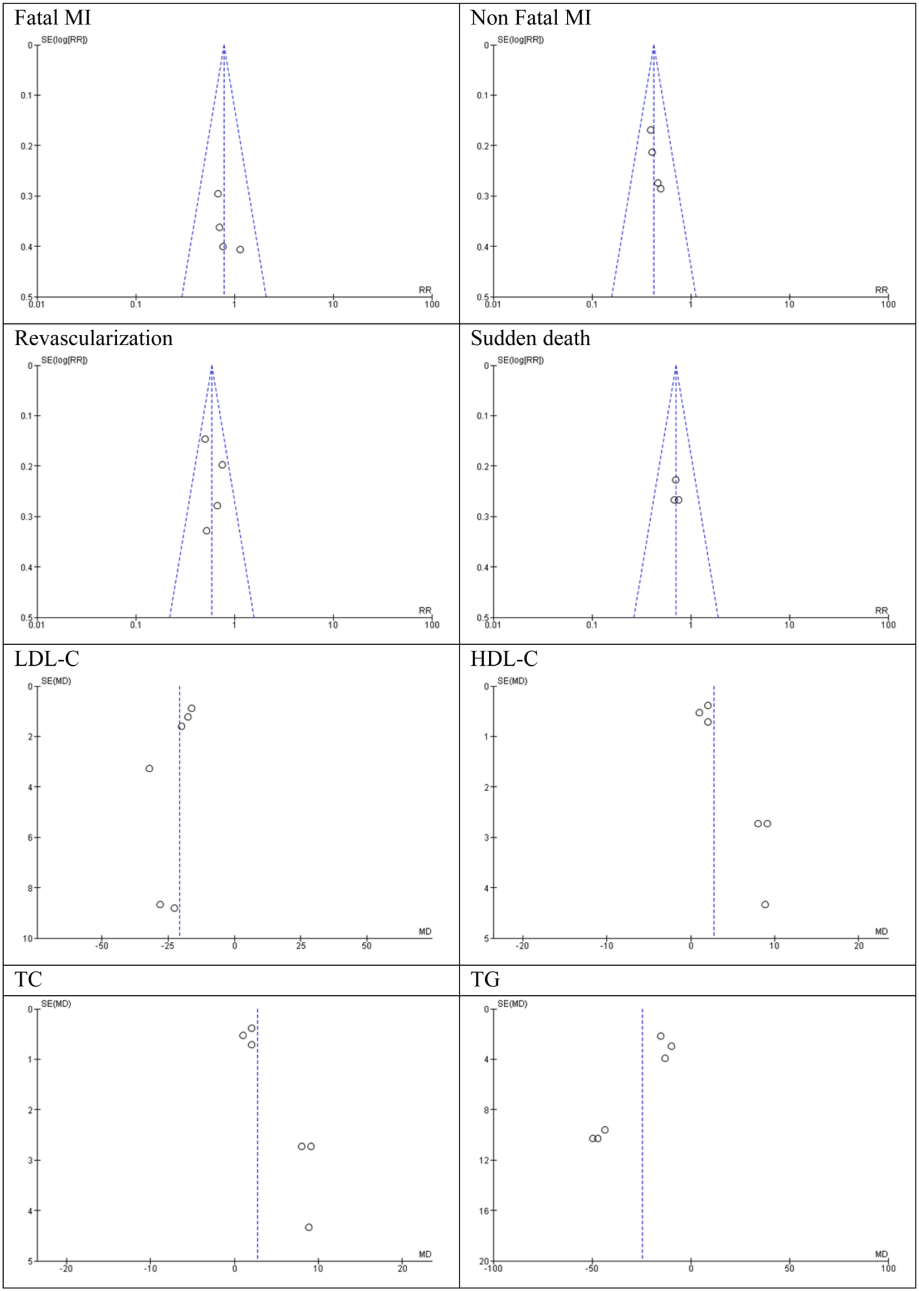
Funnel plot detailing publication bias in studies included in the meta-analysis.

The Egger’s test was used to investigate publication bias for reported LDL levels (intercept, −3.13; SE = 2.22; 95% CI −9.30 to 3.04, t = −1.41, P = 0.232), HDL levels (intercept, 3.67; SE = 1.07; 95% CI 0.71 to 6.63, t = 3.44, P = 0.026), TC levels (intercept, −3.38; SE = 2.34; 95% CI, −9.87 to 3.11, t = −1.45, P = 0.222), and TG levels (intercept, −3.81; SE = 1.55; 95% CI −8.11 to 0.49, t = −2.46, P = 0.070). Using this test, we found evidence of publication bias in HDL. We also employed funnel plots for another one of the outcomes analyzed, using visual inspection of the plots to detect publication bias. We found no evidence of bias in any of the outcomes assessed (Fig. 6).

### Sensitivity analysis

In this study we utilized the one-study remove approach. Compared to the primary analysis, the one-study remove approach indicated that changes were not found in all outcomes. Moreover, in the current study conducted analyses using both the fixed effect model and the random effect model in order to establish the sensitivity of each outcome. The results remained unchanged (Table 4).

**Table 4:** Sensitivity analysis outcomes compare main analysis.

Outcomes (References)	Main analysis	Sensitivity analysis
**Primary outcomes (N; RR (95%CI); I**^**2**^**)**		
Fatal MI^8,20-22^	10,549; 0.78 (0.55, 1.10); 0.0%	10,549; 0.78 (0.55, 1.11); 0.0%
Non Fatal MI^8,20–22^	10,549; 0.42 (0.34, 0.52); 0.0%	10,549; 0.42 (0.34, 0.52); 0.0%
Revascularization^8,20–22^	10,549; 0.58 (0.48, 0.71); 5.0%	10,549; 0.59 (0.48, 0.73); 5.0%
Sudden death^20–22^	5,679; 0.71 (0.53, 0.94); 0.0%	5,679; 0.71 (0.53, 0.94); 0.0%
**Secondary outcomes (N; WMD (95%CI); I**^**2**^**)**		
LDL^8,19–21,23,24^	9,254; −20.70 (−24.51, −16.90); 80.0%	9,254; −17.89 (−19.14, −16.64); 80.0%
HDL^8,19–21,23,24^	9,254; 2.71 (1.24, 4.17); 72.0%	9,254; 1.88 (1.33, 2.44); 72.0%
TC^8,19–21,23,24^	9,254; −26.62 (−31.65, −21.58); 90.0%	9,254; −21.36 (−22.60, −20.11); 90.0%
TG^8,19–21,23,24^	9,254; −24.74 (−34.45, −15.02); 85.0%	9,254; −15.53 (−18.58, −12.49); 85.0%

### Subgroup analysis

Subgroup analyses were conducted according to the duration of treatment. This analysis examined lipid profile outcomes in cases where the duration of treatment was either less than four years or more than four years. The results did not reveal any difference between the two groups (Table 5).

**Table 5:** Results of subgroup analysis.

Outcomes	No. of trial	Effect size	95%CI	I^2^ (%)	P for effect size	P for heterogeneity
**LDL**						
Duration (years)						
<4	3	−30.58	−36.25, −24.90	0.0	<0.00001	0.58
≥4	3	−17.58	−19.56, −15.60	52.0	<0.00001	0.12
**HDL**						
Duration (years)						
<4	3	8.61	5.17, 12.05	0.0	<0.00001	0.96
≥4	3	1.71	1.14, 2.27	21.0	<0.00001	0.28
**TC**						
Duration (years)						
<4	3	−39.61	−45.50, −33.72	0.0	<0.00001	0.59
≥4	3	−19.97	−22.89, −17.05	78.0	<0.00001	0.01
**TG**						
Duration (years)						
<4	3	−46.50	−57.86, −35.15	0.0	<0.00001	0.91
≥4	3	−13.14	−16.30, −9.98	7.0	<0.00001	0.34

## Discussion

This study constituted a systematic review and meta-analysis to determine the efficacy and safety of RYR extract for cardiovascular outcomes and lipid lowering effects in MI participants. Our meta-analysis indicated that administration of RYR extract at a dose of 1,200 mg/day could significantly improve clinical efficacy with few adverse effects, compared to placebo. The results of subgroup analyses strongly indicated clinical efficacy, an outcome which did not vary following both primary and secondary analyses.

Our findings demonstrated that RYR extract has the potential to reduce the incidence of nonfatal MI, revascularization, and sudden death, while improving lipid profiles. This finding is in agreement with the report from Shang, *et al*. that demonstrated RYR to be effective for reducing cardiovascular events in CHD patients with dyslipidemia^11^.

The mechanism whereby the incidence of nonfatal MI, revascularization, and sudden death were decreased by RYR extract remains unclear. Studies conducted in animals demonstrated that RYR increased endothelial nitric oxide synthase (eNOS) expression in vascular endothelial cells and erythrocytes, and the expression of caveolin-1 level decreasing in aorta wall. These changes are predicted to induce nitric oxide production, which was confirmed by an increase in nitrate and nitrite (NOx) levels in plasma and cGMP in the aorta wall. Histopathological study of aorta wall in rats with high cholesterol diet revealed that the development of typical plagues with macrophage infiltration was observed, while the abnormality in rats with high cholesterol diet treated with RYR were not found. In addition, the morphology of aorta wall in RYR group was also comparable to the normal control group^25^.

Moreover, RYR ameliorated oxidative stress and abnormal hemorheology, improved the pathology of atherosclerosis, and increased eNOS expression in aortic endothelium, in association with a decrease in plasma lipid levels^25,26^. A 2017 study showed that RYR extract significantly decreased oxidative stress^27^. An oxidative stress increasing expedites the progression of atherosclerosis and increases the risk of cardiovascular events by raising inflammatory reactions, endothelial dysfunction, thrombogenic tendency, plaque instability, and the migration, proliferation, and transformation of smooth muscle cells^28^. Shen *et al*. showed that RYR reduced the macrophage content in atherosclerotic lesions, consistent with plaque regression^29^. One possible mechanism proposed for the action of RYR is that RYR inhibits progression of vulnerable plaque and rupture by mitigating macrophage endoplasmic reticulum (ER) stress, consequently inhibiting apoptosis and the NF-κB pro-inflammatory pathway.

Our meta-analysis demonstrated that RYR extract significantly decreased LDL, TC, and TG. This is not surprising given that an established mechanism of RYR action is the inhibition of HMG CoA reductase^30^. Additionally, a recent study conducted in animal model found that RYR also increased the hepatic bile acids excreted, thereby increasing the need for availability of intrahepatic cholesterol used for the synthesis of additional bile^31^. Silverman found that a 1 mmol/L reduction in LDL level was associated with a 23% reduction in the risk of cardiovascular events^3^. Moreover, LDL reduction was correlated with a significant decline in the rates of myocardial infarction (MI), stroke, or coronary revascularization^32^.

The CTT analyses demonstrated the relationship between LDL reduction and cardiovascular mortality. The LDL reduction 1 mmol/L decreased 20% coronary deaths and 8% in other cardiac death^33^. However, the CTT meta-analysis found statin therapy reduced ASCVD risk but there were no associated between LDL reduction and reduction risk^34^.

In a previous meta-analysis, Li *et al*. found that RYR extract significantly decreased LDL, TC, and TG but had no effect on HDL levels^35^. Moreover, a previous report suggested that RYR reduced LDL levels significantly when compared with placebo; the effect of RYR did not differ significantly from that achieved with other lipid lowering agents^36^.

We confirmed the results of our meta-analysis by conducting a sensitivity analysis. By utilizing the one-removal approach method and changing the model to analysis of all outcomes, we found that the results remained unchanged. Therefore, our sensitivity analysis for all outcomes confirmed the robustness of our results pertaining to all outcomes.

In this meta-analysis, we synthesized all available RCT studies performing RYR treated on cardiovascular outcomes and lipid profile. The results suggest that RYR supplementation in an effective adjunct to diet therapy in borderline hypercholesterolemia patients. The current lipid management guideline^37^ recommended all patients with cardiovascular risk should be promoted a healthy life style change including exercise and diet therapy. Their reinforcement of life style change in these participants are sufficient^38^. Therefore, RYR supplementation could be a potentially alternative diet therapy.

According to the treatment duration, six weeks of RYR intervention (n = 3) has showed a significant reduction of LDL-C. As the case of statins, a reduction of LDL-C has been firstly observed within 2–4 weeks. Then, a stable LDL-C level could be found after 6 weeks of treatment^39,40^. In subgroup analysis, the result revealed that treatment at 6-week showed a better improvement than the trials with more than 4 years. Similar findings were also observed in case of HDL-C, TC, and TG. It could be explained that long term use of statin may result to drug resistance. The resistance has been related to several factors such as polymorphism of HMG-CoA reductase, P-glycoprotein, Apolipoprotein E, PCSK9, low density lipoprotein receptor (LDLR), and tumor necrosis factor α (TNF-α) genes. The resistance is probably from nonadherence to the treatment which exhibits insufficient LDL-C response to the treatment^41^.

The strength of our study is that it comprehensively summarizes the effects of RYR extract, the study being undertaken in a manner that is in accordance with a high standard of systematic review and meta-analysis, and reported in alignment with PRISMA^13^. The meta-analysis of RCT sits at the top of the hierarchy of clinical evidence. Indeed, this is the first systematic review and meta-analysis of RCT investigating efficacy and safety of RYR extract on cardiovascular outcomes and lipid profile. All of the studies included in our analysis administered RYR extract products using protocols that utilized similar laboratory analyses as well as a comparable time to follow-up (4 weeks − 4.5 years). Since there were no restrictions regarding the date or language used in the studies included in this meta-analysis, we are confident that the efficacy and safety of RYR products is quite consistent across studies. This strongly suggests that our results can be generalized to a large number of clinical practices.

## Conclusion

Based on current evidence, RYR extract therapy is predicted to be an effective and safe treatment for MI patients. However, there is insufficient data to support the hypothesis that RYP decreases the incidence of fatal MI. Therefore, well-designed, large, multi-center, randomized placebo- or active-controlled trials investigating the long term effects of RYR product therapy on MI patients are needed to further support the current evidence.
